# Endodontic therapy in a three canal mandibular second premolar

**Published:** 2006-04-01

**Authors:** Saeed Asgary

**Affiliations:** Dental Research Center, School of Dentistry, Shaheed Beheshti Medical University, Tehran, Iran

**Keywords:** Mandibular second premolar, Endodontics, Root canal morphology.

## Abstract

This article describes a clinical case of mandibular second premolar with three separated canals in apical third of the root that are diagnosed and endodontically treated. Sound knowledge of root canal anatomy and morphology, tactile examination of canal walls, critical interpretation of radiographs and high magnification examination are essential elements of success in complicated root canal therapy.

## Introduction

Complete access to and debridement of the root canal systems in endodontic treatment are key elements to long term success. Usually, the factors that make endodontic treatment unsuccessful are failures such as an inability to negotiation and incomplete instrumentation of root canals, insufficient cleaning, and poor obturation of the canals. Therefore, the clinician should have a thorough knowledge of root canal morphology and an awareness of the variations that may occur.

Endodontic treatment of mandibular premolars is one of the most difficult procedures for endodontists (1). 

Root canal morphology of mandibular premolars and the differences between first and second premolars have been investigated and reported (2-5). Mandibular second premolars had one canal at the apex in 97.5% and two canals at the apex in 2.5% of the cases (2). Studies on mandibular second premolars, however, show a low incidence of three canals. In cases their presence is noticed only after treatment, due to continuing postoperative discomfort (6). Zillich and Dowson (7) found the incidence of three canals to be 0.4%. Other studies led into similar results (8-10).

In this article an idiosyncratic case of a mandibular second premolar with three root canals was studied. It is worth noting that the three canals divided from the main canal in the apical third of the root. No previous report of a similar case was found. 

## Case report

**Figure 1:**
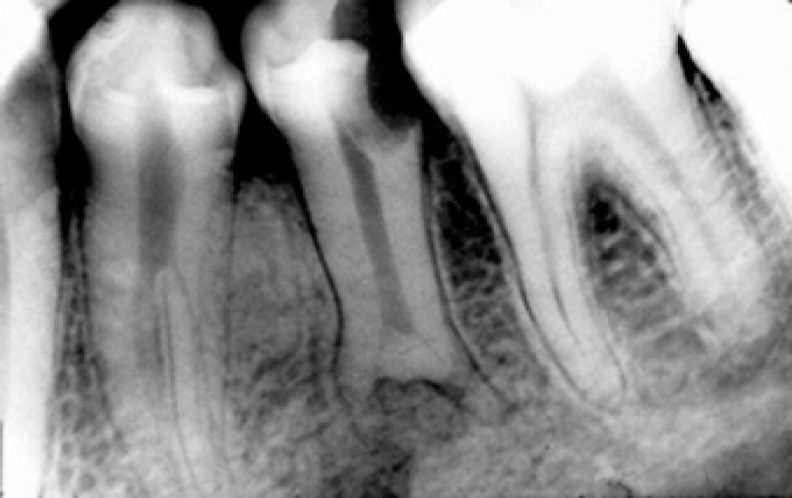
preoperative diagnostic radiograph

A 27-year-old man was referred for endodontic treatment of his mandibular left second premolar. His medical history was noncontributory. The patient reported discomfort associated with cold, hypersensitiveness, and lingering pain. His oral hygiene was poor. Clinical examination revealed several carious lesions especially on mandibular left second premolar tooth. The tooth was tender to percussion and hypersensitive to vitality tests. Preoperative radiograph showed an extensive carious lesion on tooth #20 and a sudden disappearing of the main canal in the one- third region of the root canal (Figure 1).

In the second visit, block anesthesia was administrated using 2% lidocaine with 1:80,000 epinephrine (Darupakhsh, Tehran, Iran). The tooth was isolated with rubber dam.

**Figure 2:**
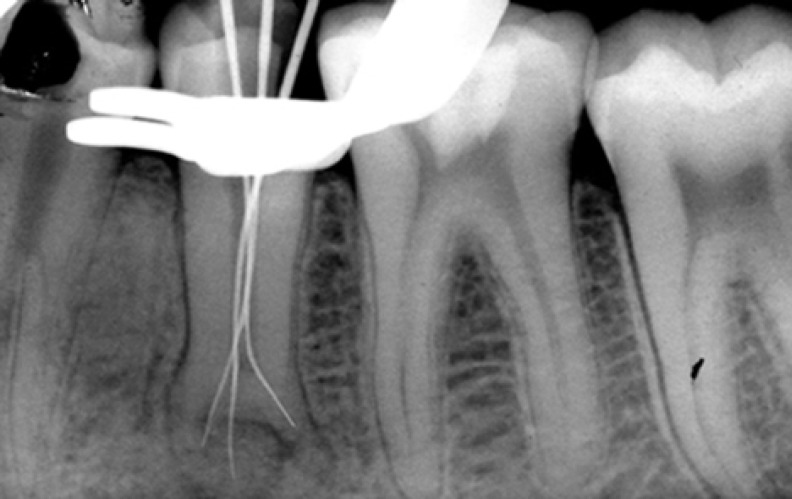
working length determination radiograph

**Figure 3:**
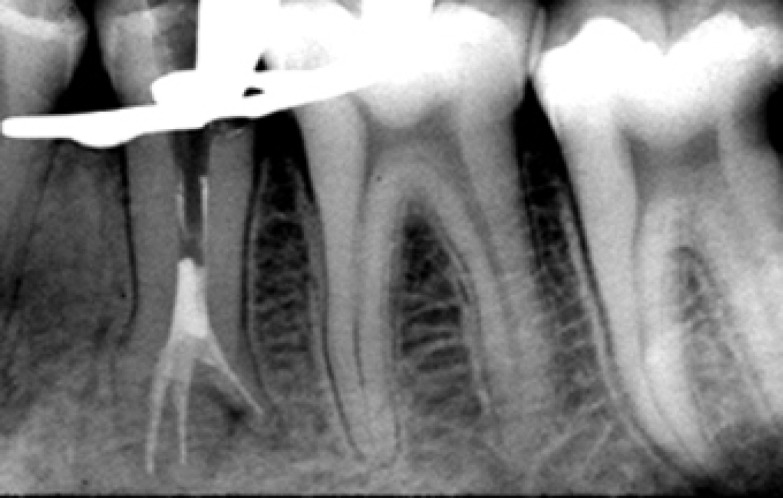
immediate postobturation radiograph

Upon complete removal of carious lesion of tooth #20, a pulp exposure due to the extensive carious process was found. Therefore the coronal access was prepared. After exact exploration a mesial canal, a buccal canal and a distolingual canal were found. The pulp was extirpated and working lengths of the canals were established (Figure 2). Normal saline solution was used for irrigation, instrumentation was completed with the final master apical file of size 30 K-flex (Mani, Japan) for all three canals and the canals were flared using step back technique. The canals were filled by lateral condensation using gutta-percha (Diadent, Korea) and Roth 801 root canal sealer (Roth international ltd., Chicago, USA) (Figure 3). This treatment was executed in a single session.

## Discussion

Mandibular premolars have highly variable root canal morphologies, but the existence of three canals is rare, especially in the second premolar. Consequently it is emphasized that, even in cases with a low probability of abnormal root canal anatomy, the existence of additional root canals has to be clinically and radiographically examined (11). A sudden narrowing of the main canal on the radiograph could be considered as a good indicator of root canal multiplicity (12) as was the starting point in the present report.
